# Curvature Determination Method for Diverging Acoustic Lens of Underwater Acoustic Transducer

**DOI:** 10.3390/s25020568

**Published:** 2025-01-19

**Authors:** Minze Li, Mingzhen Xin, Fanlin Yang, Yu Luo, Jinpeng Liu, Niuniu Wu

**Affiliations:** 1 College of Geodesy and Geomatics, Shandong University of Science and Technology, Qingdao 266590, China; 202383020109@sdust.edu.cn (M.L.); yang_navigation@yeah.net (F.Y.); luoyu@sdust.edu.cn (Y.L.); sdustljp@163.com (J.L.); wuniuniu0025@163.com (N.W.); 2 Key Laboratory of Ocean Geomatics, Ministry of Natural Resources of China, Qingdao 266590, China

**Keywords:** underwater acoustic transducer, diverging acoustic lens, acoustic emergence angle, curvature fitting, acoustic ray tracing, acoustic directivity

## Abstract

Underwater acoustic transducers need to expand the coverage of acoustic signals as much as possible in most ocean explorations, and the directivity indicators of transducers are difficult to change after the device is packaged, which makes the emergence angle of the underwater acoustic transducer limited in special operating environments, such as polar regions, submarine volcanoes, and cold springs. Taking advantage of the refractive characteristics of sound waves propagating in different media, the directivity indicators can be controlled by installing an acoustic lens outside the underwater acoustic transducer. To increase the detection range of an underwater acoustic transducer in a specific marine environment, a curvature-determining method for the diverging acoustic lens of an underwater acoustic transducer is proposed based on the acoustic ray tracing theory. The relationship equation between the original directivity indicators of the underwater acoustic transducer and the emergence angle in the specific environment is constructed, and the slope of the acoustic lens at different positions of the underwater acoustic transducer is obtained by a progressive solution. Then, the least squares polynomial fitting of the acoustic lens slope at all the refractive positions is carried out to obtain the optimal curvature of the acoustic lens. Experiments are designed to verify the effectiveness of the curvature determination method for the diverging acoustic lens of an underwater acoustic transducer, and the directivity indicators of acoustic lenses under different materials and different marine environments are analyzed. The experimental results show that the acoustic lens can change the directivity of the underwater acoustic transducer without changing the acoustic unit array, and the curvature of the acoustic lens directly affects the directivity indicators after refraction, so the method proposed in this paper has important reference value for determining the optimal shape of the diverging acoustic lens.

## 1. Introduction

The ocean accounts for 70.8% of the Earth’s surface area, and ocean exploration technology is an important foundation for the sustainable development and utilization of the ocean [1]. Under the influence of absorption, scattering, and reflection, electromagnetic waves propagating in water have the problem of rapid signal attenuation [2], and the good propagation characteristics of sound waves in seawater make underwater acoustic detection technology widely used [3,4,5]. Underwater acoustic detection systems use acoustic transducers to emit sound waves in the water and determine the position and properties of the targets by measuring the reflection, scattering, or propagation time of the sound waves [6], such as multi-beam echosounder systems (MBESs) [7,8], side-scan sonar systems (SSSs) [9], long baseline systems (LBL) [10], short baseline systems (SBL) [11], ultra-short baseline systems (USBL) [12] et al. The directivity indicators of such acoustic systems mainly depend on the array arrangement of the acoustic units in the underwater acoustic transducer, and the control of the sound wave directivity is realized through the planar or non-planar array arrangement. On the one hand, the range of the array size and emergence angle adjustment will be physically limited, resulting in the sound wave directivity not being able to cover the required range in some cases. On the other hand, underwater acoustic transducers are expensive to design, manufacture, and package, and once the array is packaged, the sound wave directivity is fixed and cannot be adapted to different marine environments.

An acoustic lens is a device used to control the propagation direction of sound waves to improve the resolution and sensitivity of acoustic signals by controlling the propagation direction and energy distribution of sound waves. Acoustic lenses make use of the refractive nature of sound waves in different media and can adjust the propagation characteristics of sound waves, such as focusing, scattering, and refraction, by designing the shape, density, and other parameters of the lens. At present, acoustic lenses have been widely studied and applied in many fields [13,14,15,16,17,18]. Kim et al. [19] designed an adaptive acoustic lens for sound fire extinguishers to improve the fire extinguishing effect in various fire fighting environments by improving the beam convergence performance of sound fire extinguishers. Stephens et al. [20] designed a circular cross-sectional acoustic lens for imaging catheters to assist intracardiac echocardiography (ICE) in forming the best beam shape so as to realize intracardiac ultrasound imaging visualization and synchronize the 3D position information. Tol et al. [21] used Luneburg lenses with hexagonal unit cells of different diameters to control elastic waves and verified the effect of the omnidirectional focusing ability of Luneburg lenses in improving the directional sensitivity of energy harvesters. In a study of marine acoustic lenses, Sato et al. [22] designed a non-planar Fresnel acoustic lens and numerically calculated the convergent acoustic pressure field of the lens by using the two-dimensional finite difference time domain method to improve the resolution of underwater imaging. Ding et al. [23] analyzed the acoustic wave convergence performance of single-concave acoustic lenses and double-concave acoustic lenses and improved the transmitted voltage response level and overall electroacoustic conversion efficiency of the transducer in underwater communication by optimizing the beam directivity of the planar piston directional transducer. Huang et al. [24] improved a new composite acoustic lens with a specific unit cell combination of cross-shaped structures to achieve underwater 3D focusing by increasing the acoustic field intensity at the focal point of the acoustic lens.

The directivity indicators of the fixed array underwater acoustic transducer are precisely calibrated under laboratory conditions, and the directivity indicators are directly related to the detection range and efficiency. However, the temporal and spatial variation in non-uniform water may cause the directivity indicators of the transducer to decrease greatly in actual ocean exploration, especially in extreme marine environments such as polar regions, submarine volcanoes, and cold springs, so it is a feasible solution to adjust the directivity of the transducer by using acoustic lenses. To solve the problem of the limited acoustic signal coverage of an underwater acoustic transducer in ocean exploration, this paper carries out research work around the diverging acoustic lens of underwater acoustic transducers to achieve the emergence angle improvement of underwater acoustic transducers in a specific ocean environment. The main contributions are as follows: (1) the refraction effect of an underwater acoustic transducer under an acoustic lens is modeled based on the acoustic ray tracing theory; (2) a curvature determination method for the diverging acoustic lens of an underwater acoustic transducer is proposed, which solves the problem of determining the optimal shape of the acoustic lens when the emergence angle is known; (3) the proposed method and the performance of the acoustic lens in regard to changing the directivity indicators are analyzed and discussed through experiments.

The organizational structure of this paper is as follows: In Section 2, the refractive principle of the diverging acoustic lenses is expounded based on acoustic ray tracing. In Section 3, the method of determining the curvature and the optimal shape of the acoustic lens is derived. In Section 4, experiments are designed to verify the curvature determination method of the diverging acoustic lens of an underwater acoustic transducer, and the influences of factors such as emergence angle, acoustic lens material, and marine environment changes are analyzed and discussed. In Section 5, the research work of this paper is summarized.

## 2. Principle of Diverging Acoustic Lens Refraction

**Figure 1:**
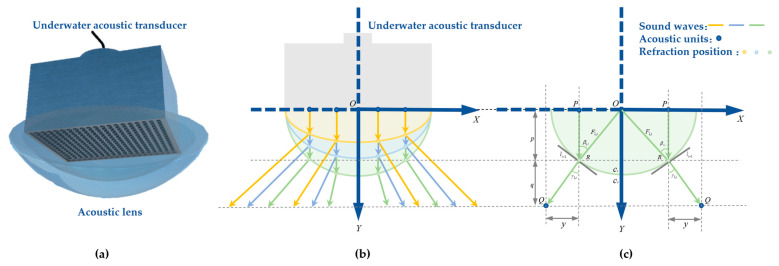
Schematic diagram of the diverging acoustic lens of an underwater acoustic transducer. (**a**) Underwater acoustic transducers and acoustic lenses; (**b**) propagation of sound waves in acoustic lenses; (**c**) Snell’s law of sound propagation.

The working principle of the underwater acoustic transducer is to use the piezoelectric effect, magnetoelectric effect, acousto-optic effect, and other physical phenomena of the material to realize the conversion of underwater acoustic signals and electrical signals. The underwater acoustic transducer can be designed with various shapes and sizes according to the application requirements, such as the planar array, cylindrical array, and spherical array. This paper utilizes an underwater acoustic transducer with a planar array as an example to analyze. As shown in Figure 1a, the sound wave directivity indicators of the array are fixed after the array package is completed and cannot be adjusted adaptively for different marine environments. Therefore, the directivity indicators can be adjusted by installing diverging acoustic lenses on the outside of the underwater acoustic transducer. As shown in Figure 1b, the sound wave is refracted at the interface between the acoustic lens and seawater after the installation of a diverging acoustic lens, so the directivity indicators of the sound wave can be adjusted by controlling the curvature of the acoustic lens.

The acoustic ray tracing theory is widely used in the study of sound wave directivity. As shown in Figure 1c, the sound wave propagates from medium 1 into medium 2 so that the propagation speed of the sound wave in medium 1 is $\;c_{1}$ and the propagation speed in medium 2 is $\;c_{2}$ The time $\;t_{s}$ required for the sound wave to travel from the launching point P to the target point Q is described as

(1) $$t_{s}=\frac{\sqrt{x^{2}+p^{2}}}{c_{1}}+\frac{\sqrt{y^{2}+q^{2}}}{c_{2}}$$

where  x  is the horizontal distance between the launching point P and the refraction point R,  y  is the horizontal distance between the target point Q and the refraction point R,  p  is the vertical distance between the launching point P and the refraction point R, and  q  is the vertical distance between the target point Q and the refraction point R.

According to Fermat’s principle, finding the shortest time from the launching point P to the target point Q is defined as

(2) $$\frac{dt_{s}}{dx}=\frac{x}{c_{1}\sqrt{x^{2}+p^{2}}}-\frac{y}{c_{2}\sqrt{y^{2}+q^{2}}}$$

(3) $$\frac{d^{2}t_{s}}{dx^{2}}=\frac{p^{2}}{c_{1}{\left(x^{2}+p^{2}\right)}^{2/3}}+\frac{q^{2}}{c_{2}{\left(y^{2}+q^{2}\right)}^{2/3}}$$

If $\frac{d^{2}t_{s}}{dx^{2}}>0$, and $\frac{dt_{s}}{dx}<0\left(x=0\right)$, and $\frac{dt_{s}}{dx}>0\left(x=y\right)$, it can be obtained that $\frac{dt_{s}}{dx}=0$ exists at $x=x_{0},x_{0}\in \left(0,y\right)$, then it is defined as

(4) $$\frac{x}{c_{1}\sqrt{x^{2}+p^{2}}}-\frac{y}{c_{2}\sqrt{y^{2}+q^{2}}}=0$$

Then, the Snell’s law can be defined as

(5) $$\rho =\frac{\sin\beta_{1,0}}{c_{0}}=\frac{\sin\beta_{1,1}}{c_{1}}\ldots =\frac{\sin\beta_{1,i}}{c_{i}}=\frac{\sin\beta_{1,i+1}}{c_{i+1}}$$

where the Snell constant is ρ. The incidence angle is $\beta_{1,i}$, which was defined as the angle between the propagation direction of the sound wave at depth $z_{k}$ and the normal or vertical direction. When the incidence angle and the sound velocity profile are known, the incidence angle at any depth of water can be determined by Snell’s law, which also means that the propagation direction of the sound wave is determined. Therefore, when the inherent directivity indicators of the underwater acoustic transducer, the propagation speed of the sound wave in the acoustic lens, and the propagation speed of the sound wave in the seawater are known, if the emergence angle of underwater acoustic transducer is given, the curvature of the acoustic lens can be optimally fitted and calculated so as to achieve the purpose of expanding the detection range of the underwater acoustic equipment.

## 3. Curvature Determination Method for Diverging Acoustic Lens

(1) Constructed coordinate system

The key of the diverging acoustic lens is to construct the relationship equation between the original directivity indicators of the underwater acoustic transducer and the emergence angle in a specific environment. The O coordinate system is constructed based on the central position of the acoustic unit $P_{i}\left(i=1,2,\cdots ,n\right)$ of the underwater acoustic transducer, which reflects the relative position distribution of the acoustic unit in the device.

(2) We calculated the position of the refractive point and the tangent over this point.

The coordinate position of the acoustic unit $P_{i}$ in the O coordinate system is $\left(x_{p_{i}},y_{p_{i}}\right)$ and the incidence angle $\beta_{1,i}$ can be defined as

(6) $$\beta_{1,i}=\arcsin\left(\rho_{1,i}c_{1}\right)=\arcsin\left(\sin\gamma_{1,i}c_{1}/c_{2}\right)$$

where is $\rho_{1,i}$ the Snell constant between the diverging acoustic lens layer and the seawater layer, $c_{1}$ is the propagation speed of the sound wave in the diverging acoustic lens layer, $c_{2}$ is the propagation speed of the sound wave in the seawater layer, and $\gamma_{1,i}$ is the refracted emergence angle of the sound wave at the junction of the diverging acoustic lens layer and the seawater layer, which can be expressed as

(7) $$\gamma_{1,i}=\arcsin\left(c_{2}\cdot \sin\left(\beta_{1,i}\right)/c_{1}\right)$$

When the $i^{th}$ acoustic unit emits a sound wave through the diverging acoustic lens layer, the straight line connecting the refraction point R at the junction between the diverging acoustic lens layer and the seawater layer to the coordinate origin O is $F_{1,i}$, the slope of this line is $L_{i}\_k$ and the intercept is $L_{i}\_b$, so the linear equation $Y_{L_{i}}$ is defined as

(8) $$Y_{L_{i}}=L_{i}\_k\cdot X_{L_{i}}+L_{i}\_b$$

where $\omega_{1.i}$ is the angle between the vertical direction and the horizontal direction, the slope is $L_{i}\_k=\tan\left(\beta_{2,i}\right)$, and the angle between the line connecting the refracted point R to the coordinate origin O is $\beta_{2,i}=\omega_{1,i}-\beta_{1,i}$.

Then, the equation corresponding to the tangent line $l_{i}$ of the refraction position is expressed as

(9) $$Y_{l_{i}}=l_{i}\_k\cdot X_{l_{i}}+l_{i}\_b$$

where the tangent slope of the $i^{th}$ acoustic unit refracted at the position of the diverging acoustic lens layer is $l_{i}\_k=\tan\left(2\beta_{1,i}+\beta_{2,i}-\alpha_{i}\right)$, the intercept is $l_{i}\_b$, and $\alpha_{i}$ is the angle between the straight line $F_{1,i}$ and the tangent line $l_{i}$.

The refractive position $R_{i}$ of the $i^{th}$ acoustic unit in the diverging acoustic lens layer along the maximum emergent direction can be defined as

(10) $$\left\{\begin{array}{c} x_{i}=\frac{L_{i}\_b-l_{i-1}\_b}{l_{i-1}\_k-L_{i}\_k}\\ y_{i}=L_{i}\_k\cdot x_{i}+L_{i}\_b \end{array}\right.$$

(3) Curvature determination and shape fitting

The curvature at $R_{i}$ is defined as

(11) $$K_{i}=\frac{\left|l_{i}\_k^{\prime }\right|}{{\left(1+l_{i}\_k^{2}\right)}^{\frac{3}{2}}}$$

The polynomial model can be defined as

(12) $$f\left(x_{i}\right)=a_{0}+a_{1}x_{i}+a_{2}x_{i}^{2}+\cdots +a_{m}x_{i}^{m}=\sum_{j=0}^{m}a_{j}x_{i}^{j}$$

where m is polynomial order, and $a_{j}\left(j=1,2,\cdots ,m\right)$ is the polynomial coefficient.

The equation set is constructed as

(13) $$\left\{\begin{array}{c} f\left(x_{1}\right)=a_{0}+a_{1}x_{1}+a_{2}x_{1}^{2}+\cdots +a_{m}x_{1}^{m}\\ f\left(x_{2}\right)=a_{0}+a_{1}x_{2}+a_{2}x_{2}^{2}+\cdots +a_{m}x_{2}^{m}\\ \vdots \\ f\left(x_{n}\right)=a_{0}+a_{1}x_{n}+a_{2}x_{n}^{2}+\cdots +a_{m}x_{n}^{m} \end{array}\right.$$

And the matrix form of Equation (13) is

(14) BL=G

where B is the coefficient matrix, L is the unknown parameter vector, and G is observation vector, They can be expressed as follows

$$B=\left(\begin{array}{ccccc} 1 & x_{1} & x_{1}^{2} & \cdots & x_{1}^{m}\\ 1 & x_{2} & x_{2}^{2} & \cdots & x_{2}^{m}\\ \vdots & \vdots & \vdots & \cdots & \vdots \\ 1 & x_{n} & x_{n}^{2} & \cdots & x_{n}^{m} \end{array}\right),\;L=\left(\begin{array}{c} a_{0}\\ a_{1}\\ \vdots \\ a_{m} \end{array}\right),\;G=\left(\begin{array}{c} f\left(x_{1}\right)\\ f\left(x_{2}\right)\\ \vdots \\ f\left(x_{n}\right) \end{array}\right)$$

Then, the least squares solution is

(15) $$L={\left(B^{T}B\right)}^{-1}B^{T}G$$

**Figure 2:**
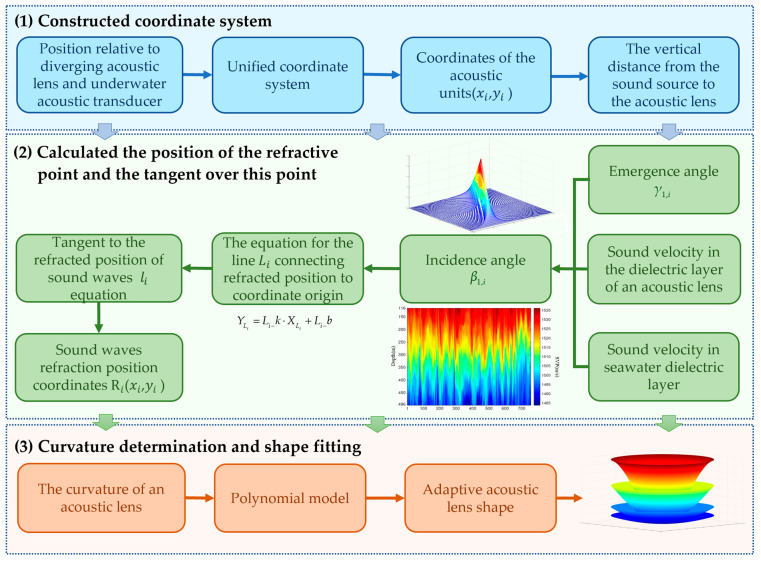
Curvature determination algorithm flow chart.

And the optimal shape of the diverging acoustic lens can be fitted. The specific curvature determination algorithm is shown in Figure 2.

## 4. Experiments and Analysis

Table 1 below shows the overall planning contents and parameter settings of the experiments. It mainly consists of four experiments; experiment 1 is the validation of the effectiveness of the curvature determination method for the diverging acoustic lens, experiment 2 is the influence analysis of the emergence angle, experiment 3 is the influence analysis of materials for making acoustic lenses, and experiment 4 is the influence analysis of marine environmental change.

### 4.1. Validation of the Effectiveness of the Curvature Determination Method for Diverging Acoustic Lens

**Figure 3:**
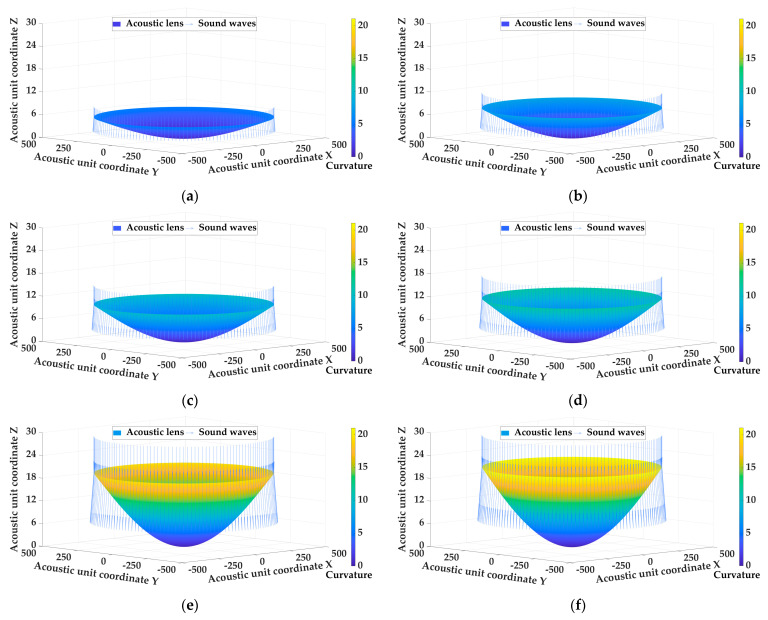
The shape of the acoustic lens with the different materials. (**a**) PDMS; (**b**) LK; (**c**) SI; (**d**) RTV; (**e**) LDPE; (**f**) PS.

Experiments were designed to verify the curvature determination method for diverging acoustic lens of underwater acoustic transducer. Common acoustic lenses materials were used, such as polydimethylsiloxane (PDMS), kerosene (LK), silicone resin (SI), room-temperature vulcanized silicone (RTV), low-density polyethylene (LDPE), and polystyrene (PS). Assuming that the underwater acoustic transducer was placed in a constant marine environment with a temperature of 25 °C, a salinity of 33.622 PUS, and a pressure of 65.745 dbar, then the propagation speed of the sound wave through different materials and seawater can be determined. The emergence angle of the underwater acoustic transducer was set to be 45° after the diverging acoustic lens was installed to determine the optimal fitting shape of the acoustic lens using different materials. Table 2 shows the fitting curvature distribution of the diverging acoustic lens with different materials and Figure 3 shows the shape of the acoustic lens with the different materials.

As shown in Figure 3 and Table 2, when the material of the acoustic lens is determined, the curvature determination method for the diverging acoustic lens of the underwater acoustic transducer is used to determine the shape of the acoustic lens that can meet the requirements of the emergence angle by calculating the slope of the sound wave at the junction of the lens layer and the seawater layer. The shape of the acoustic lens determined by different materials is quite different. The maximum curvature value of the acoustic lens obtained by PS material fitting is 21.002 m^−1^, while the maximum curvature value obtained by PDMS material fitting is 5.418 m^−1^, which is directly due to the different propagation speed of sound waves in different materials. Therefore, there are many factors that affect the fitting shape of the acoustic lens, including the demand for the emergence angle, the material of the acoustic lens, and the change in the marine environment et al., and the influence of these factors on the curvature fitting and shape determination of the acoustic lens is analyzed in the next step.

### 4.2. Influence Analysis of Emergence Angle

**Figure 4:**
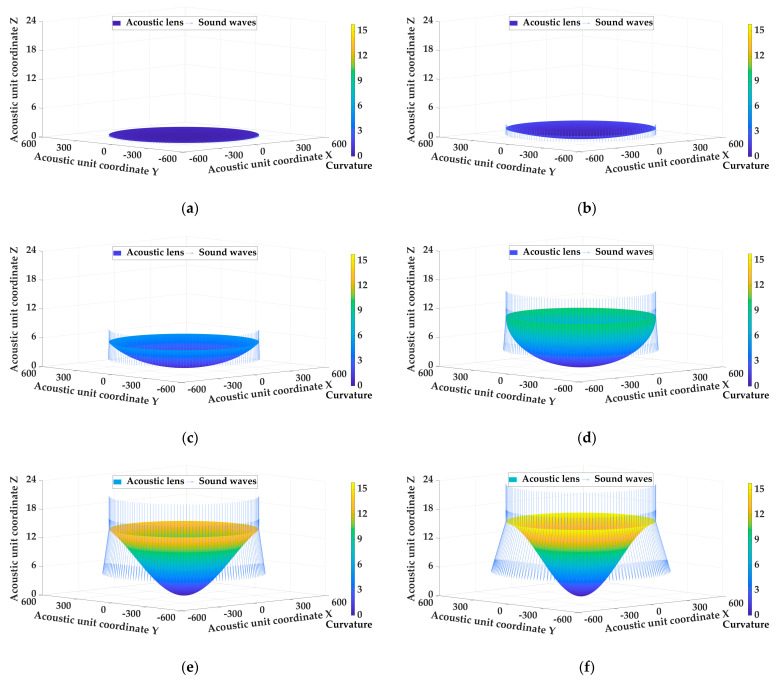
The shape of the acoustic lens with the different emergence angles. (**a**) Emergence angle = 10°; (**b**) emergence angle = 20°; (**c**) emergence angle = 35°; (**d**) emergence angle = 55°; (**e**) emergence angle = 70°; (**f**) emergence angle = 85°.

When designing acoustic lenses, the emergence angle is an important directivity indicator. A larger emergence angle can increase the measurement range and allow the underwater acoustic transducer to detect a wider area. Assuming that the underwater acoustic transducer was placed in a constant marine environment with a temperature of 25 °C, a salinity of 33.622 PUS, a pressure of 65.745 dbar, and the material was LK, the emergence angle of the underwater acoustic transducer was used as a variable factor to evaluate the influence of the emergence angle. Table 3 shows a fitting curvature distribution of the diverging acoustic lens with different emergence angles, and Figure 4 shows the shape of the acoustic lens with the different emergence angles.

Combined with Figure 4 and Table 3, the influence on the emergence angle is analyzed as follows:

(1) When the marine environment and the material of the acoustic lens are constant, the measurement range of the acoustic lens expands with the increase in the emergence angle. However, the emergence angle should not be increased indefinitely, as too large of an emergence angle may reduce the resolution of the target, making it difficult to distinguish between different targets or subtle marine terrain and organisms.

(2) As the emergence angle increases, the curvature of the acoustic lens increases accordingly. When the emergence angle is 10°, the curvature of the acoustic lens changes less obviously, the maximum curvature is 0.473 m^−1^, and the average curvature is 0.156 m^−1^. When the emergence angle is 85°, the curvature of the acoustic lens changes significantly, the maximum curvature is 15.584 m^−1^, and the average curvature is 7.392 m^−1^.

(3) The increase in the emergence angle leads to a higher convexity of the fitted acoustic lens shape, which directly affects the refractive characteristics of the sound wave. However, the increase in convexity will increase the difficulty of making acoustic lens, so the relationship between the emergence angle and the surface curvature of the acoustic lens needs to be fully considered in the design process to achieve a more efficient underwater detection range.

### 4.3. Influence Analysis of Materials for Making Acoustic Lenses

**Figure 5:**
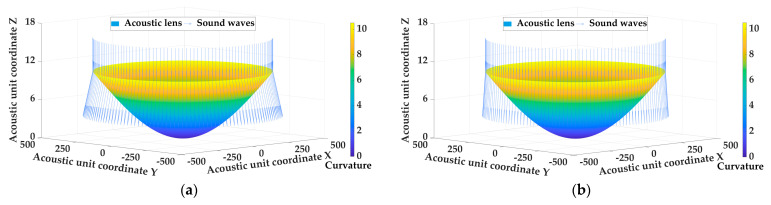

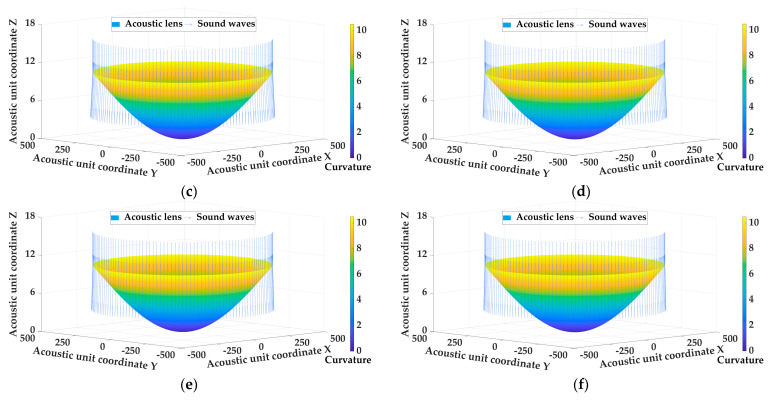
The variation in the maximum emergence angle of the acoustic lens with different materials. (**a**) PDMS; (**b**) LK; (**c**) SI; (**d**) RTV; (**e**) LDPE; (**f**) PS.

Assuming that the underwater acoustic transducer was placed in a constant marine environment, and that the fitting shape of the acoustic lens was determined, the variation in the emergence angle of the acoustic lens with different materials was analyzed. Using the curvature determination method for the diverging acoustic lens of an underwater acoustic transducer, the maximum emergence angle of the acoustic lens with different materials can be obtained. Table 4 shows the maximum emergence angle of the acoustic lens with different materials, and Figure 5 shows the variation in the maximum emergence angle of the acoustic lens with different materials.

Combined with Figure 5 and Table 4, the analysis of the effects on the acoustic lens material is as follows:

(1) The greater the difference between the sound speed of the acoustic lens material and the sound speed of seawater, the more significant the change in the maximum emergence angle. When the sound speed of the material used is less than that of seawater, the sound wave propagates more slowly and the maximum emergence angle increases significantly so that the measurement range is increased accordingly. When the sound speed of the material is higher than the sound speed of seawater, the sound wave propagates faster in it and the maximum emergence angle does not increase significantly.

(2) Among all the acoustic lens materials used, PDMS has good plasticity, high-and low-temperature resistance and chemical stability, and the emergence angle of PDMS acoustic lens has the highest increase ratio, reaching 79%. PS has good acoustic noise reduction characteristics, but the emergence angle of the PS acoustic lens has the lowest improvement ratio, reaching 27%.

(3) The propagation speed of acoustic waves in the acoustic lens material has a direct impact on the emergence angle; therefore, when selecting the acoustic lens material, it is necessary to consider not only the ability to improve the emergence angle but also the plasticity and stability of the actual application requirements so as to ensure that the underwater acoustic transducer can obtain accurate and reliable measurement results within the required range.

### 4.4. Influence Analysis of Marine Environmental Change

**Figure 6:**
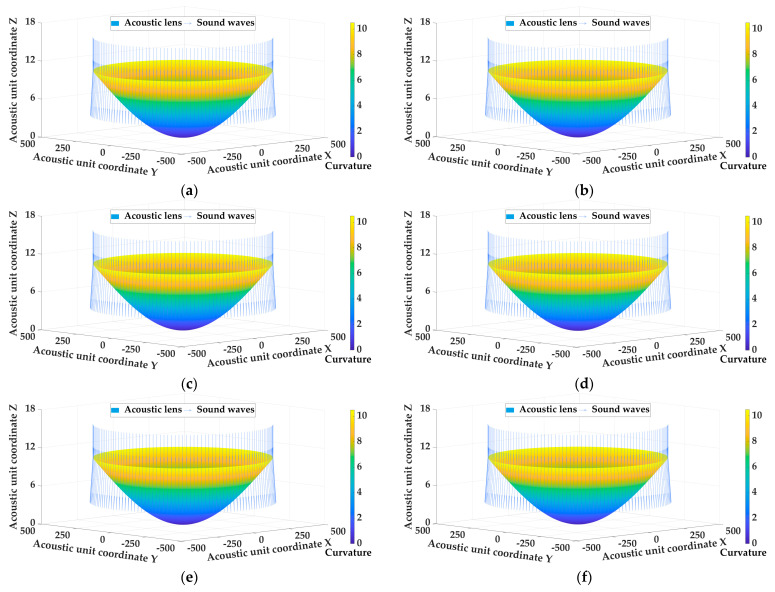
The variation in the maximum emergence angle of the acoustic lens with different marine environments. (**a**) T = −2 °C, C = 1440 m/s; (**b**) T = 5 °C, C = 1485 m/s; (**c**) T = 10 °C, C = 1497 m/s; (**d**) T = 16 °C, C = 1512 m/s; (**e**) T = 23 °C, C = 1527 m/s; (**f**) T = 30 °C, C = 1546 m/s.

Assuming that the fitting shape of the acoustic lens and the material (LK) of the acoustic lens were determined, the propagation speed of sound waves in seawater was changed and the effects of different marine environments were evaluated. Using the curvature determination method for the diverging acoustic lens of an underwater acoustic transducer, the maximum emergence angle of the acoustic lens with different marine environments can be obtained. Table 5 shows the maximum emergence angle of the acoustic lens with different marine environments and Figure 6 shows the variation in the maximum emergence angle of the acoustic lens with different marine environments.

Combined with Figure 6 and Table 5, the impact on changes in the marine environments is analyzed as follows:

(1) In the case of a certain material and shape of the acoustic lens, the propagation speed of the sound wave in the seawater will have an effect on the emergence angle. The sound speed of seawater is a function of temperature, salinity, and pressure, and the temperature change has the most direct effect on the sound speed of seawater.

(2) In the case of relatively high marine environmental temperatures, the propagation speed of sound waves in seawater increases and the maximum emergence angle increases, for example, the maximum emergence angle is 55°39′23.32″ in the marine environment of 30 °C. In the case of a relatively low marine environmental temperature, the propagation speed of sound waves in seawater decreases and the maximum emergence angle becomes smaller; for example, the maximum emergence angle is 50°17′24.61″ in the marine environment of −2 °C.

(3) Therefore, the changes in the marine environment at a low temperature, normal temperature, and high temperature will affect the performance of the acoustic lens. For the design and application of the acoustic lens, it is necessary to consider the influence of the change in ocean sound speed, as well as the response characteristics of the acoustic lens material to the temperature to ensure the stability and reliability of its performance in different marine environments.

## 5. Conclusions

The irregular changes in the complex marine environment will affect the propagation path of a sound wave, causing the phenomena of refraction, scattering, and attenuation, which will weaken the transmission efficiency and measurement range of acoustic signals. An underwater acoustic transducer often needs to maximize the coverage of acoustic signals, but most of the transducer directivity indicators are difficult to change after the device is packaged. In special operating environments, such as polar regions, submarine volcanoes, and cold springs, the emergence angle of the underwater acoustic transducer will be limited. Based on the acoustic ray tracing theory, a curvature determination method for the diverging acoustic lens of an underwater acoustic transducer is proposed, the relationship equation between the original directivity indicators of the underwater acoustic transducer and the emergence angle is constructed, the slope of the acoustic lens at the refraction position is taken as an unknown parameter for the least squares solution, and the optimal shape of the acoustic lens is finally fitted. The experimental results show that the method proposed in this paper can realize the curvature fitting of the diverging acoustic lens, the acoustic lens can change the directivity indicators of the underwater acoustic transducer without changing the acoustic array, and the acoustic lens fitted by this method can greatly improve the emergence angle. By optimizing the curvature of the acoustic lens, the measurement range of the underwater acoustic transducer can be extended and the measurement accuracy and efficiency can be further improved. An acoustic lens can improve the flexibility and sustainable utilization of an underwater acoustic transducer, so it has important research value and application prospects.

## Figures and Tables

**Table 1:** The overall contents and parameter setting of the experiment.

Experiments	Materials	Emergence Angles	Temperatures
1 Curvature determination experiment	PDMS, LK, SI RTV, LDPE, PS	45°	25 °C
2 Emergence angles experiment	LK	10°, 20°, 35° 55°, 70°, 85°	25 °C
3 Materials experiment	PDMS, LK, SI RTV, LDPE, PS	/	25 °C
4 Marine environmental experiment	LK	/	−2 °C, 5 °C,10 °C 16 °C, 23 °C, 30 °C

**Table 2:** The fitting curvature distribution of the diverging acoustic lens with different materials.

Materials	Sound Speed (m/s)	Curvature Range (m^−1^)	Median Curvature (m^−1^)	Mean Curvature (m^−1^)
PDMS	1100	0~5.418	1.600	1.970
LK	1324	0~7.849	2.318	2.854
SI	1485	0~9.874	2.916	3.591
RTV	1610	0~11.628	3.439	4.235
LDPE	2080	0~19.372	5.739	7.068
PS	2340	0~21.002	6.046	7.541

**Table 3:** The fitting curvature distribution of the diverging acoustic lens with different emergence angles.

Emergence Angles (°)	Curvature Range (m^−1^)	Median Curvature (m^−1^)	Mean Curvature (m^−1^)
10	0~0.473	0.117	0.156
20	0~1.834	0.431	0.589
35	0~5.165	1.033	1.509
55	0~10.529	3.371	4.007
70	0~13.864	5.206	5.789
85	0~15.584	7.219	7.392

**Table 4:** The maximum emergence angle of the acoustic lens with different materials.

Materials	Sound Speed (m/s)	Maximum Emergence Angle
PDMS	1100	79°47′23.29″
LK	1324	54°51′4.07″
SI	1485	46°48′12.60″
RTV	1610	42°15′12.11″
LDPE	2080	31°21′49.95″
PS	2340	27°33′27.62″

**Table 5:** The maximum emergence angle of the acoustic lens with different marine environments.

Marine Temperature/°C	Sound Speed (m/s)	Maximum Emergence Angle
−2	1440	50°17′24.61″
5	1485	52°30′57.79″
10	1497	53°06′09.44″
16	1512	53°52′19.74″
23	1527	54°38′20.68″
30	1546	55°39′23.32″

## Data Availability

Data are contained within the article.
